# Performing at the Top of One's Musical Game

**DOI:** 10.3389/fpsyg.2016.01356

**Published:** 2016-09-13

**Authors:** Johannes L. Hatfield

**Affiliations:** Music Education, Norwegian Academy of MusicOslo, Norway

**Keywords:** instrumental practice, psychological skills, self-regulation, goal setting, motivation, perfectionism, self-efficacy

## Abstract

The purpose of the present mixed method study was to investigate personal benefits, perceptions, and the effect of a 15-week sport psychological skills training program adapted for musicians. The program was individually tailored for six music performance students with the objective of facilitating the participants' instrumental practice and performance. The participants learnt techniques such as *goal setting, attentional focus, arousal regulation, imagery*, and *acceptance training*/*self-talk*. Zimmerman's (1989) cyclical model of self-regulated learning was applied as a theoretical frame for the intervention. The present study's mixed-method approach (i.e., quan+ QUAL) included effect size, semi-structured interviews, a research log, and practice diaries of the participants (Creswell, 2009). Thematic analysis revealed that participants had little or no experience concerning planning and goal setting in regard to instrumental practice. Concentration, volition, and physical pain were additional issues that the participants struggled with at the time of pre-intervention. The study found that psychological skills training (with special emphasis on planning and goal setting) facilitated cyclical self-regulated learning patterns in the participants. In essence, the intervention was found to facilitate the participants' concentration, self-observation, self-efficacy, and coping in the face of failure. The appliance of practice journals facilitated the participants‘ self-observation, self-evaluation, and awareness of instrumental practice. Finally, the psychological skills intervention reduced participants' worry and anxiety in performance situations. An 8-month follow up interview revealed that the participants were still actively applying psychological skills.

## Introduction

What does the number one tennis player Roger Federer have in common with the world famous pianist Leif Ove Andsnes? Intuitively, one may believe they represent two completely different phenomena. However, digging more profoundly into this matter, we realize they are both performers expected to achieve at the highest level. Both performers have achieved their level of success through winning competitions, thus overcoming tremendous external and internal pressure. Moreover, their attainment of expertise is based on thousands of hours of deliberate practice ensuing tremendous motor control. In comparison to aspiring musicians, aspiring athletes are to a greater extent supported by a huge apparatus of coaches, doctors and physical therapists. This enables the best possible training and performance conditions for each individual athlete (Hays, 2002). Talented musicians, however, are to a great extent left to their own devices. They spend most of their time practicing their instrument individually in a practice room (Lehmann and Jørgensen, 2012; Burwell and Shipton, 2013). Although music students are involved in playing chamber music as well as symphonic music in higher music education, ensemble playing constitutes just a minor part of a total amount of practice. Accumulating 7800 individual practice hours throughout 5 years of music studies is not uncommon (Jørgensen, 1996). Studying music on the highest level is all about becoming as skilled as possible (Lehmann and Ericsson, 1997). Such one-dimensional striving for perfection and excellence makes music college students more disposed to maladaptive perfectionism than non-music college majors (Wang and Zhao, 2009)^1^. Stoeber and Eismann (2007) found that young musicians striving for perfectionism (i.e., *perfectionistic strivers*) were associated with intrinsic motivation, high performance standards, and deliberate effort. Musicians who were concerned with perfectionism (i.e., *perfectionistic concerners*) were associated with controlled motivation, performance anxiety, somatic complains, and emotional fatigue. Research also reveals that music students have a perception of being taught *what* to practice rather than *how* to practice (Jørgensen, 2000; Atkins, 2009; Gaunt, 2009; Burwell and Shipton, 2013). The violinist, Norbert Brainin of the Amadeus String Quartet describes his relationship with his teacher, Carl Flesch like “*that of a doctor and patient. Flesch would listen, diagnose whatever your problems were, and suggest a remedy which would have the merit of helping you to help yourself to improve”* (Snowman, 1981, p. 16). Evidently, Flesch saw the importance of teaching his students *how* to practice giving prescriptions of further exploration and improvement. Providing athletes with the best possible path to athletic excellence is not solely based on deliberate physical training, but psychological training as well (Starkes and Ericsson, 2003; Weinberg and Gould, 2011). In essence, physical and psychological training is viewed as inextricable entities (Hanrahan and Andersen, 2010; Weinberg and Gould, 2011). Both Federer and Andsnes have spent considerable hours on mental/psychological training as part of their training and practice. However, Federer was introduced to psychological skills training due to his training program (Stauffer, 2006). Andsnes reports that he explored new ways of working mentally as a result of foregoing struggle in his instrumental practice during his early twenties (based on my conversation with Andsnes). Moreover, it is believed that young aspiring musicians, similarly to aspiring athletes, would benefit from having the possibility of learning psychological skills. Psychological skills training is believed to advance the quality of motivation, self-awareness and self-regulation in musicians, preventing burnout, procrastination and injuries along the way (Fournier et al., 2005; Lemyre et al., 2005). However, surprisingly few studies have tried out psychological skills training in the context of music acquisition and performance (Clark and Williamon, 2011; Hoffman and Hanrahan, 2012; Osborne et al., 2014).

The primary aim of the present study was to investigate personal benefits, perceptions, and the effects of an individually tailored psychological skills training intervention for performing music students. Zimmerman's cyclical model of self-regulation was applied as both the theoretical and practical frame of the intervention (Zimmerman and Schunk, 1989; Zimmerman, 2002).

### Psychological skills training and self-regulated learning

*Psychological skills training* (PST) is a “*systematic and consistent practice of mental or psychological skills for the purpose of enhancing performance, increasing enjoyment, or achieving greater sport and physical self-satisfaction”* (Weinberg and Gould, 2011, p. 248). Principles deriving from PST have been developed and applied successfully in the realm of sports during the last five decades (Orlick and Partington, 1988; Sheard and Golby, 2006; Hays, 2009; Thelwell et al., 2010; Weinberg and Gould, 2011; Beauchamp et al., 2012; Papnikolaou et al., 2012). PST includes *goal setting, attentional focus, arousal regulation, imagery, and acceptance training/self-talk*^2^. Foundationally, goal setting is considered the core psychological technique within PST (Mellalieu and Hanton, 2009; Weinberg and Gould, 2011). Research on goal setting in sports has found personal, specific, and short-term goals the most effective in training and competition (Burton, 1989). In comparison to long-term goals, specific short-term goals contain the quality of specifically defining what, how and why to carry out one's training on micro level (Burton, 1989). In their méta analysis, Kyllo and Landers found that specific short-term goals are more effective when hierarchically combined with long-term goals (Kyllo and Landers, 1995). In essence, hierarchal goal setting provides information and rationales gauging the learning process (i.e., long-term goals nourish medium, and short-term goals) (Locke and Latham, 2002; Zimmerman, 2008). Locke et al. (1981) found that *specific* and *difficult* goals were more effective than very *easy*, very *hard*, and “*do you best*” goals. Easy goals are commonly attributed as boring and thus unsatisfying, while very difficult goals seem to be interpreted as unrealistic and thus demoralizing (Locke et al., 1981). Moreover, providing aspiring athletes with challenging goals based on realistic achievement levels facilitates athletes' (tentatively musicians') effort and motivation (Burton, 1989; Weinberg and Gould, 2011).

**Figure 1:**
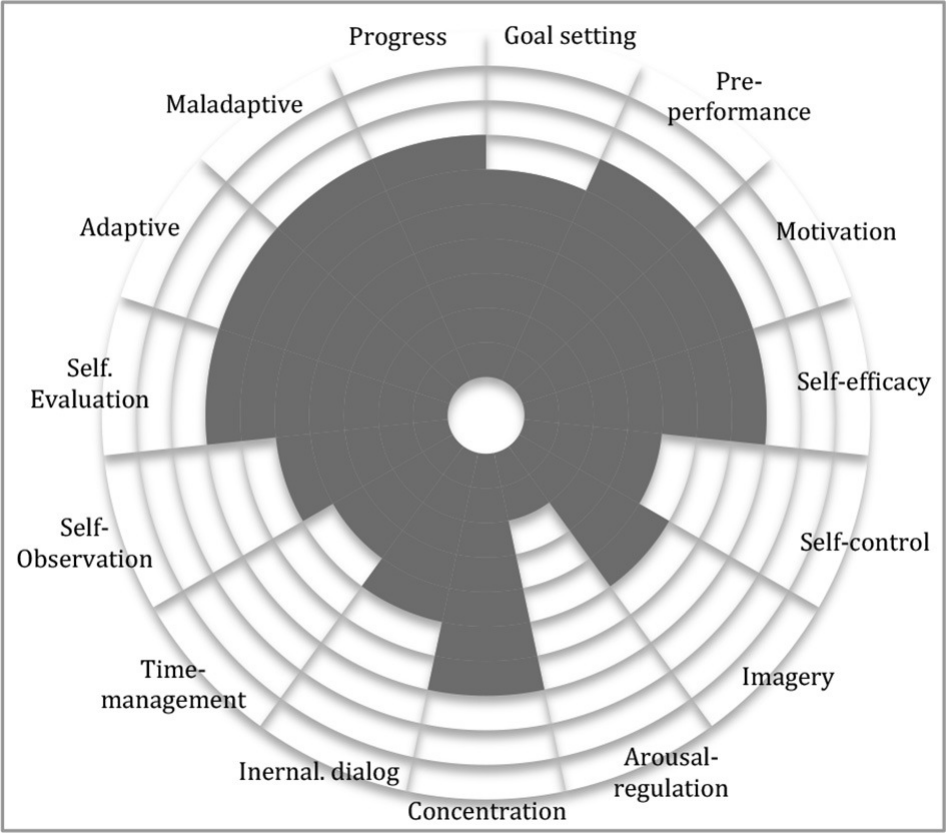
**Performance profile illustrating strengths in gray and limitations in white**.

**Figure 2:**
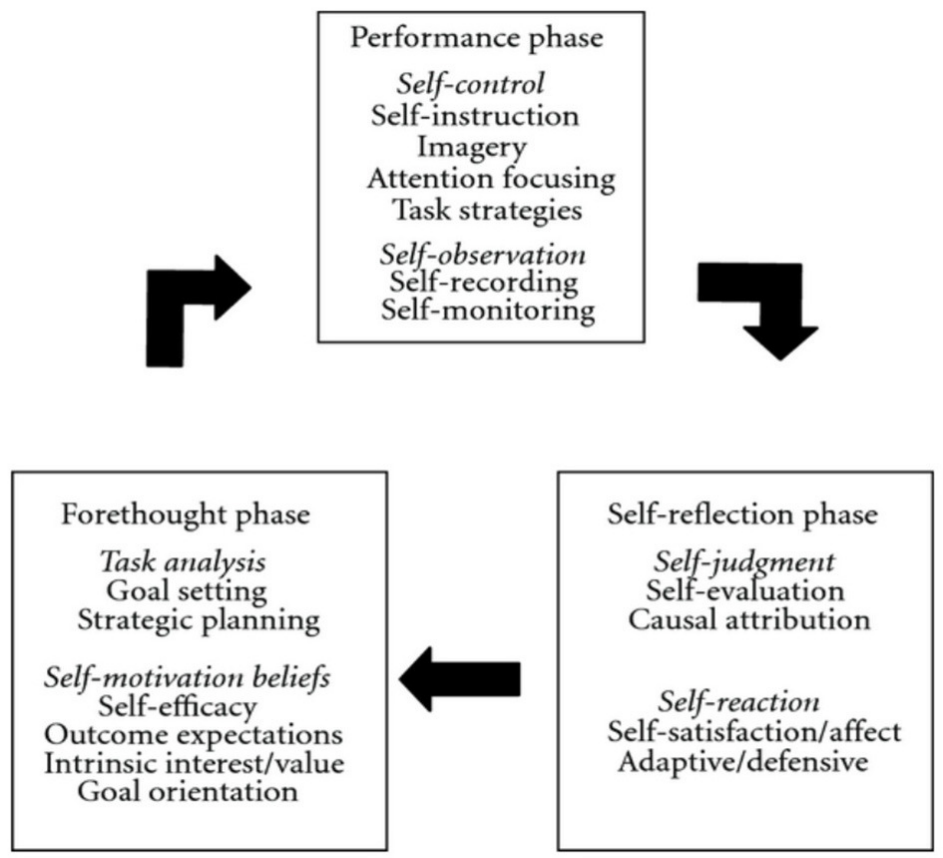
**Zimmerman's cyclical model of self-regulated learning (Zimmerman, 2002)**.

Self-Regulated Learning (SRL) refers to “*self-generated thought, feelings, and actions that are oriented to attaining goals. These learners are proactive in their efforts to learn because they are aware of their strength and limitations and because they are guided by personally set goals and task related strategies”* (Zimmerman, 2002, p. 66). Similarly to SRL, strengths and limitations are the first aspects assessed in the initial phase of PST (Andersen, 2000, 2005; Hays, 2009). In supporting initial assessment, *performance profiling* (i.e., through which both coaches and athletes assess and rank strengths and limitations in athletes) aids subsequent goal setting and strategic planning (Figure 1; Weinberg and Gould, 2011; Hatfield and Lemyre, 2016). In strategic planning, athletes apply training journals for systemizing and defining how, when, and why training is being exerted. Notes concerning potential goal-adjustments are written down based on continuous self-evaluation (Andersen, 2000, 2005; Hays, 2009). Zimmerman's (1989) theory of self-regulation explicates processes of learning cyclically (Locke, 1968; Bandura, 1977; Nicholls, 1984; Weiner, 1985; Zimmerman and Schunk, 1989; Zimmerman et al., 1992). Zimmerman's cyclical model (Figure 2) entails three reciprocal phases: forethought, performance and self-reflection phases. “*Forethought processes precede efforts to learn and are designed to enhance those efforts. Performance phase processes occur during learning efforts and are designed to improve action and self-monitoring. Self-reflection processes occur after learning efforts and are designed to optimize a person's reactions to his or hers outcomes”* (Zimmerman, 2008, p. 287).

Studies regarding self-regulation in instrumental practice have found that self-regulated music students were metacognitive, self-efficacious, gritty, and proactive in regard to instrumental practice. Furthermore, these studies have found that self-regulated music students have a larger repertoire of practice strategies than less self-regulated music students (Nielsen, 2004; Leon-Guerrero, 2008; McPherson et al., 2013; Miksza and Tan, 2015; Hatfield et al., 2016). Applying structural equation modeling, Hatfield et al. (2016) tested Zimmerman's (1989) cyclical model of self-regulated learning among 204 music students. The study found that self-regulated music students approached their instrumental practice cyclically. The present study applied Zimmerman's cyclical model (Figure 2) as the theoretical lens for both the implementation and interpretation of PST. Forethought phase processes emphasized deliberate individual assessment and self-referenced goals as a foundation for continuity and progress. The performance phase included psychological skills such as attentional focus, arousal-regulation, imagery, and acceptance training/internal dialogue, as well as self-observation. In relation to the self-reflection phase of Zimmerman's cyclical model, participants were encouraged to apply music practice journals for ongoing reflection and evaluation of the individual work carried out (for review see Figure 2).

### Intervention research in the music field

Research in performance sciences is growing. The field has investigated instrumental practice quality, self-regulation, and deliberate practice for decades (Ericsson et al., 1993; Jørgensen and Lehmann, 1997; Lehmann and Ericsson, 1997; McPherson and Renwick, 2001; Hallam, 2001; Nielsen, 2001, 2004, 2008; Jørgensen, 2008; Miksza, 2009; Lehmann and Jørgensen, 2012; McPherson et al., 2013; Miksza and Tan, 2015). However, few studies have investigated practice quality, self-regulation, and deliberate practice from an interventional standpoint (Clark and Williamon, 2011; Osborne et al., 2014; Hatfield and Lemyre, 2016). Clark and Williamon (2011) tried out a 9-week mental skills intervention for music students at The Royal College of Music in London. Quantitative findings revealed a significant increase in self-efficacy, imagery vividness, and enhancement of instrumental practice behaviors. Significant changes in cognitive, somatic anxiety, and self-confidence were not found. The qualitative findings revealed an increase in self-confidence, motivation, goal setting and self-awareness.

Retrospective feedback regarding the overall program revealed that participants requested a greater use of group interaction and discussions, thus learning from one another. Better and more frequent application of skills in the performance situation was also requested (Clark and Williamon, 2011). Hatfield and Lemyre (2016) piloted a 2-month PST-intervention assessing the efficacy of various intervention tools. Findings revealed that the PST-program benefited from taking an individual person-based approach. Supporting each participant's psychological needs enhanced self-awareness and intrinsic motivation for PST. The appliance of electronic practice journals affected the participants' self-observation, goal setting and self-evaluation in a positive way. The study also found that socializing through group sessions benefited participants' need for relatedness. The group sessions turned out to provide the participants with a quasi-concert situation in which they had the opportunity to apply the psychological skills (Hatfield and Lemyre, 2016). A 3-week performance psychology program among advanced music students revealed a significant increase in participants' confidence, courage, focus, concentration, resilience and preparation from pre- to post-testing. However, the program failed to show significant increase in mental toughness, energy regulation and optimal energy (Osborne et al., 2014). Accordingly, two short-term intervention studies implementing techniques deriving from cognitive behavioral therapy (CBT) showed a decrease in perceived performance anxiety and an increase in performance quality (Kendrick et al., 1982; Hoffman and Hanrahan, 2012). All the above studies (except Clark and Williamon, 2011; Hatfield and Lemyre, 2016) were short-term studies providing mental/psychological training to participants in groups. According to applied sport psychology literature, short-term interventions with a non-individual focus might be subject to some limitation (Hays, 2009). Similarly to learning any complex skill, PST requires both time and effort (Andersen, 2000, 2005; Hays, 2009). Other types of interventions such as yoga (Khalsa and Cope, 2006), Alexander technique (Valentine et al., 1995), have revealed positive effects in reducing performance anxiety in musicians. Khalsa and Cope (2006) found that yoga had some positive significant impact on reducing high levels of stress, performance-related musculoskeletal conditions, and performance anxiety. Valentine et al. (1995) found that Alexander technique enhanced musical and technical quality of performance to some extent. Some reduction in anxiety and less variation in heart rate was also observed in the experimental group. These findings underscore the importance of body awareness and arousal regulation in regard to practicing and performing music. Lastly, *mental rehearsal* in the field of music is a widely evaluated mental skill that aims at enhancing music performance (Bird, 1984; Ross, 1985; Coffman, 1990; Driskell et al., 1994; Gregg and Clark, 2007; Haddon, 2007; McHugh-Grifa, 2011). Generally, most of this research has found that the combination of mental and physical practice leads to the best result (Ross, 1985; Driskell et al., 1994; McHugh-Grifa, 2011). This line of research has mainly covered mental rehearsal in terms of mental rehearsal of a music score. Through reviewing mental rehearsal in sports and music, Gregg and Clark (2007) call for a broader, and more structured approach to mental rehearsal in music. Accordingly, the present study aims to apply imagery in terms of both memorizing positive outcomes and preparing emotionally for performance (for review see Smith et al., 2007).

Research individually tailoring PST to musicians is underrepresented in the field of performance sciences. Thus, in contrast to previous studies (e.g., Hoffman and Hanrahan, 2012), the present study took a long-term individually based approach to PST. The overall educational aims of the PST were to: Automate and overlearn the skills, systematically integrate PST in performance situations, and simulate the skills in various performance-related contexts (Weinberg and Gould, 2011).

## Research questions

The present study was based on the following research questions:

1. How can PST facilitate music students' instrumental practice and performance?
2. Might the use of psychological skills have a positive impact on music students' perceived *self-efficacy, motivation*, and satisfaction with progress and performance?
3. Might the use of psychological skills decrease music student's *anxiety* and *worry* in instrumental practice and performance?

Since the program constituted an *individual* approach, the participants were expected to develop differently throughout the intervention according to individual needs and the personal work.

## Methods and materials

The present study was based on a pilot study that aimed *to “build foundations on how to implement future interventions to guide music students on how to optimize practice toward performance”* (Hatfield and Lemyre, 2016, p. 1). The pilot study found individualized approaches to work sufficiently as a foundation for PST. Furthermore, the pilot study found intervention tools such as questionnaires, performance profiling, practice journals, combined group and individual sessions, as well as self-referenced goals to benefit the efficacy of PST-interventions for musicians (Hatfield and Lemyre, 2016). Expanding on the pilot study, the present study included refined versions of the intervention tools assessed in the pilot study (i.e., performance profiling, self-reference, iPads/electronic practice journals, and use of a combination of group and individual meetings)^3^.

### Participants and procedures

During the second half of the fall semester 2014, six music performance majors were voluntarily recruited from the music academy's performance grogram. Due to a high interest rate, an electronic questionnaire was developed for screening the most suitable cases. The questionnaire was sent to all the 26 potential recruits assessing their availability, previous experience with PST, and degree of interest in PST^4^. Following the selection process, six participants were selected for the study. For anonymity purposes, all six participants were given the abbreviation S1–S6 (S = Student). The final case sample included two jazz performance students (winds, S1 and S6) and four classical performance students (3 strings, 1 woodwind, S2, S3, S4, and S5). All the participants attended the Academy's bachelor program. After completing the recruitment process, a consent form approving full anonymity and volunteering was provided. One week prior to the intervention, the participants were sent the Self-regulated Learning in Music Questionnaire (SLMQ). The participants were interviewed during the first individual session. The assessment routine was repeated at post-intervention assessing individual progress throughout the program. Eight months after completing the intervention, participants were sent a written based follow-up interview assessing their current use of PST. The Norwegian Social Science Data Service approved the study and its procedures.

### The psychological skills training program

The PST-intervention lasted for 15 weeks, from 12th of January until 27th of April 2015. The PST-intervention included 15 individual sessions and 7 group sessions (i.e., the group sessions took place every other week followed up by a 30 min individual session. The group sessions lasted for 60–90 min, while the remaining 8 individual sessions lasted for about 60 min). All participants were asked to pick at least two works of music they wanted to study throughout the intervention (i.e., from scratch to the concert podium). The participants were told to perform their selected works both in concert and in two dress rehearsals. An individual approach laid the basis for the overall implementation and development of the PST-intervention. In essence, self-assessment (i.e., including SLMQ and pre-intervention interviews) laid the foundation for individual tailoring of PST. The individual sessions worked as an arena for elaborating individual needs and training procedures. The group sessions encouraged the participants to apply the techniques they were working on in front of their colleagues. The group sessions also encouraged participants to exchange ideas and experiences. Hence, the participants learnt and applied PST both individually and together with other participants. Internalizing and frequently trying out the psychological skills was highlighted throughout the intervention. In coherence with sport psychological conventions, goal setting constituted a foundation for ongoing PST and individual progress. Based on individual needs, the students worked on techniques including attentional focus/concentration (e.g., centering), imagery, arousal-regulation, and acceptance training (e.g., self-talk) (Andersen, 2000, 2005; Hayes and Strosahl, 2004; Hays and Brown, 2004; Russel, 2006; Harris, 2009; Hays, 2009; Weinberg and Gould, 2011; Hatfield and Lemyre, 2016). The main objective was to give the participants a “hands on experience” throughout the program (i.e., working with the instrument in hand giving the participants as much practical experience as possible).

The first author delivered all group and individual PST sessions. In addition to having studied sport science/psychology and educational science, the first author had years of training and experience as a professional cellist. Moreover, the combination of extensive knowledge of PST and instrumental practice/performance enabled the application of appropriate integration and adaption of PST-techniques (Andersen, 2000, 2005; Hays, 2002, 2009).

### Quantitative measures

Self-regulated Learning in Music Questionnaire (SLMQ), Hatfield et al. (2016) was applied assessing the participants' strengths and limitations in self-regulated learning. For a detailed measurement review see (Hatfield et al., 2016). All scores were recorded on a five-point Likert scale ranging from (1) “strongly disagree” to (5) “strongly agree.” The questionnaire was structured in accordance with the three phases in Zimmerman's cyclical model of self-regulated learning (see Figure 2; Zimmerman, 2002).

#### The forethought phase

*The goal setting scale* (6 items, α = 0.80) measured students' use of goal setting, emphasizing specific and hierarchical goals (e.g., “in relation to my long term goals, I set specific short term goals for my practice”). The *Self-efficacy scale* (4 items, α = 0.77) measured self-efficacy for instrumental practice (e.g., “I strongly believe that I have what it takes to accomplish what I start working on”). *The time-management* scale (3 items, α = 0.73) measured to what extent music the music students managed the time of their overall practice and time of each practice session (e.g., “I follow a well developed plan for how long I should practice”). *The worry scale* (4 items, α = 0.74) measured the students' internal and external worry of failure while performaing (e.g., “I often think to myself, “what if I am not prepared enough for this performance”).

#### Performance phase

*The self-observation scale* (3 items, α = 0.74) measured to what degree the students were involved in self-monitoring. Moreover, checking one's quality, precision, and use of metacognition while practicing (e.g., “I observe my practice from an analytical perspective”).

*The self-control scale* (3 items, α = 0.63) measured to what extent the music students stick to deliberate ways of practicing (e.g., “I tend to lose focus toward tasks while practicing due to a desire to master the task immediately”). *The concentration scale* (3 items, α = 0.64) measured to what extent the music students mange to direct their focus toward task relevant activities while practicing (e.g., “It is easy for me to direct my focus toward what I am practicing”). *The imagery scale* (2 items, α = 0.87) measured to what extent the music students apply imagery in relation to instrumental practice and music performances (e.g., “I use imagery in relation to instrumental practice”). *The arousal regulation* scale (3 items, α = 0.58) measured mental and physical arousal (reversed items) (e.g., “I often get overly tense during concerts and badly influenced by this”).

#### Self-reflection phase

*The self-evaluation scale* (3 items, α = 0.73) measured the students' involvement in the evalution of their instrumental practice (e.g., “When having practiced something during longer periods, I look back to see if I did the right procedures”). *The coping scale* (3 items, α = 0.69) measured to what extent the music students find new ways and strategies in the face of failure (e.g., “When I'm not achieving the desired results, I carefully search for plausible reasons leading to new more adequate goals”). *Perception of progress* was measured with one single item (i.e., “I believe that my current progress reflects the amount of hours spent on practicing”).

Generally, the internal consistency was very good, except arousal-regulation (α = 0.58), self-control (α = 0.63), concentration (α = 0.64), and coping (α = 0.69), which according to conservative criteria (Kerlinger, 1974) were acceptable. The four items were deemed acceptable and kept accordingly (for review see Hatfield et al., 2016).

### Qualitative measures

1. *Semi-structured interviews* were carried out in relation to pre- and post-testing. Due to the present study's individual focus, the interviews were divided into two sections, a general (i.e., consisting of the same questions) and an individualized section (i.e., based on individual answers from the questionnaire).
2. A *research log*, assessing individual and group meetings, was applied reporting each participant's progress throughout the intervention. Information including the participants‘ and the researcher's perceptions of individual work, development, and progress was documented on a weekly basis in the log.
3. The participants' *practice journals* documenting personal goals, self-reflection, self-evaluation, and pre- to post-test development triangulated the data collection.
4. Based on Hatfield and Lemyre (2016), *Mini-iPads* were handed out to each participant. The iTune-based application MusicJournal was pre-installed in all the iPads. Besides writing their practice journals, the participants applied the iPads for recording and assessing individual progress.

## Analysis

The present study applied mixed methods (i.e., concurrent nested design), “*mixed methods research is research design (or methodology) in which the researcher collects, analyzes, and mixes (integrates or connects) both quantitative and qualitative data in a single study or multiphase program of inquiry”* (Creswell and Plano Clark, 2007, p. 119). The quantitative method supplemented the qualitative methods. Within methods triangulation, which “*serves to clarify meaning by identifying different ways the phenomenon is being seen”* (Stake, 1995, p. 214), was applied in relation to the qualitative data (i.e., semi-structured interviews, research log, and participants practice journals).

### Quantitative analysis

Kolmogorov-Smirnov criteria for normal distribution (K-S) found that 9 of the 13 sub-scales met the test criteria for normal distribution (*p* > 0.5). Consequently, a paired sample *T*-test was applied measuring the 9 sub-scales meeting the K-S criteria, (*p* > 0.5). Wilcoxon signed ranks test (i.e., a non-parametric equivalent to paired sample *T*-test) measured the remaining 4 scales. Hedges G illustrated standardized effect size measures^5^ (Hedges and Olkin, 1985).

### Qualitative analysis

Qualitative data was analyzed based on principles deriving from thematic analysis (Braun and Clarke, 2006; Guest et al., 2012). In accordance with thematic analysis, all qualitative data was carefully analyzed in the following stages (Braun and Clarke, 2006):

1. Familiarization with data supported by transcription and repeated reading of the raw data making initial ideas and impressions.
2. Initial coding based on both theory (i.e., self-regulation theory) and identified relevant features systematically identified throughout the data set.
3. Generating potential themes based on initial codes linking all relevant data.
4. Reviewing to what extent the themes generated fit the coded extracts and the overall data set.
5. Defining and naming of themes in relation to the overall story deriving from the analysis.
6. Producing the report selecting vivid extract examples and final analysis related to the research question and the literature creating the report.

Data from all three sources (i.e., semi-structured interviews, research log, practice journals) were in the end integrated into comprised case reports including key themes representing each participant's development throughout the program. Key themes were subsequently compared across cases, reported, and illustrated through thematic mapping (Braun and Clarke, 2006).

## Results

### Quantitative findings

The quantitative component in the present study was meant to supply and clarify the qualitative aspects, which served as the main empiric body of the present research (i.e., concurrent nested design) (Creswell, 2009).

#### Effects of the program

**Figure 3:**
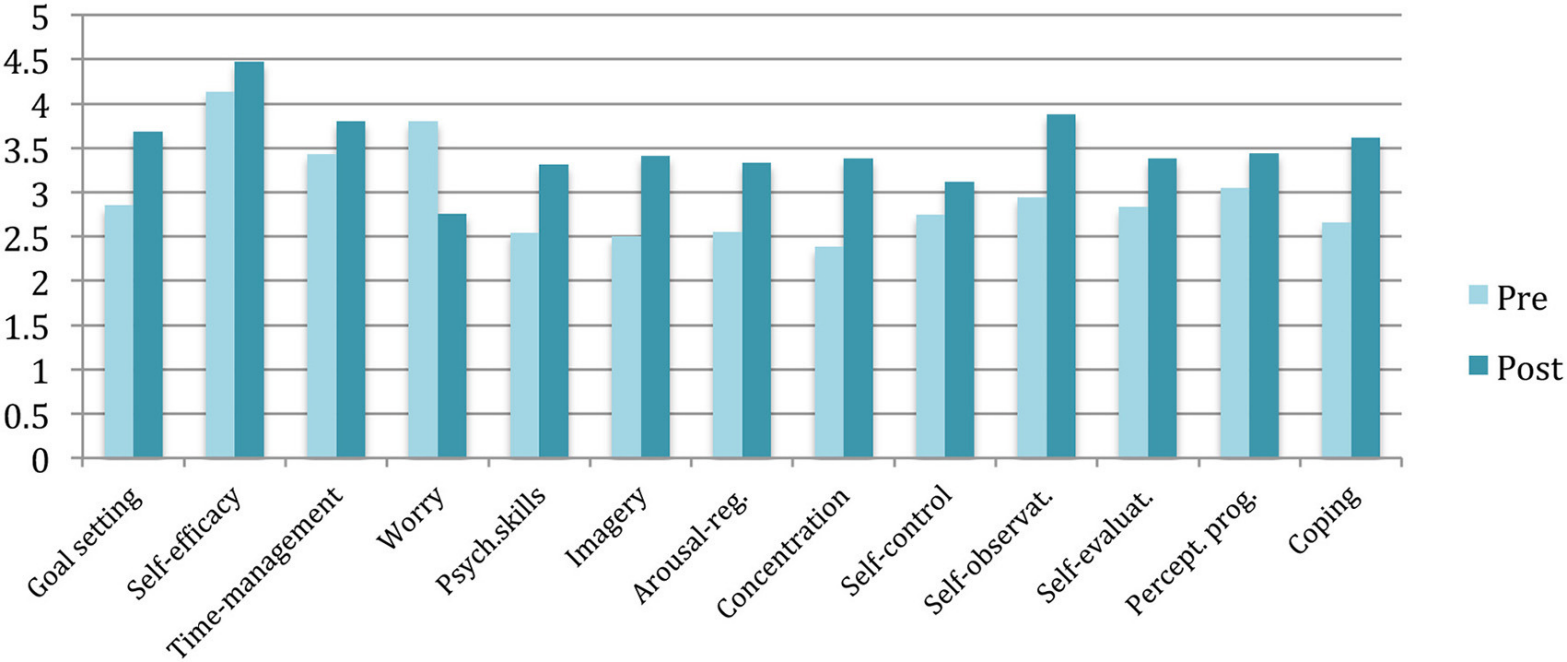
**Diagram illustrating group means from pre- to post-intervention**.

As illustrated in Tables 1, 2, the quantitative measures revealed a significant increase in the use of psychological skills such as goal setting, imagery, arousal-regulation, concentration, and self-observation. Wilcoxon signed rank test revealed that self-reflection processes such as self-evaluation and coping increased significantly from time 1 to time 2. The participants' worry decreased significantly from pre- to post-testing. Figure 3 illustrates a general increase in all the variables (i.e., except worry) from pre- to post-intervention. Individual mean measures are illustrated in Table 3 showing each participant's quantitative development. The measures generally reveal (with some exceptions) increase in pre- to post-test mean measures.

**Table 1:** **SLMQ-subscale means (and SD) pre- and post-program with paired sample *T*-test**.

**SLMQ subscales**	**Time 1 Mean (*SD*)**	**Time 2 Mean (*SD*)**	***t***	***p***	***g***
Goal-setting	2.85 (0.59)	3.68 (0.65)	4.90^**^	**0.00**	1.2
Self-efficacy	4.13 (0.60)	4.47 (0.35)	1.58	0.17	0.63
Time-management	3.43 (1.0)	3.80 (0.94)	1.21	0.28	0.35
Psych. skills:	2.54 (0.27)	3.31 (0.25)	8.45^***^	**0.00**	2.7
Imagery	2.50 (1.0)	3.41 (0.97)	3.05^*^	**0.02**	0.85
Arousal-regulation	2.55 (0.77)	3.33 (0.63)	2.76^*^	**0.04**	1.0
Concentration	2.38 (0.68)	3.38 (0.57)	4.74^***^	**0.00**	1.4
Self-control	2.75 (0.63)	3.12 (0.34)	1.32	0.24	0.67
Self-observation	2.94 (0.38)	3.88 (0.54)	9.22^***^	**0.00**	1.8

* *p < 0.05*,

** *p < 0.01*,

*** *p < 0.001*.

*Degrees of freedom for all t-tests are 5. g = Hedges' g standardized effect size measures*.

*The bold values indicate statistically significant effect size measures*.

**Table 2:** **SLMQ-subscale means (and SD) pre- and post-program with Wilcoxon signed-rank test**.

**SLMQ subscales**	**Time 1 Mean (*SD*)**	**Time 2 Mean (*SD*)**	***Z***	**Wilc. sig**.	***g***
Worry	3.80 (0.70)	2.76 (0.62)	−2.20	**0.028**	−1.4
Self-evaluation	2.83 (1.0)	3.38 (0.87)	1.84	**0.066**	0.054
Perception of prog.	3.05 (1.1)	3.44 (0.40)	0.730	0.456	0.43
Coping	2.66 (0.66)	3.61 (0.40)	2.33	**0.020**	1.6

*The significance level, < 0.05 was increased to < 0.10, adjusting for insufficient power (Pallant, 2013)*.

*The bold values indicate statistically significant effect size measures*.

**Table 3:** **SLMQ—individual subscale means for pre- and post-testing**.

**SLMQ subscales**	1^*^	2^*^	3^*^	4^*^	5^*^	6^*^	7^*^	8^*^	9^*^	10^*^	11^*^	12^*^	13^*^
Student 1 pre	2.50	4.17	2.00	4.00	2.29	2.50	2.67	2.00	2.00	3.33	2.00	3.67	2.67
Student 1 post	3.00	4.00	2.20	2.80	3.19	4.00	3.33	2.67	2.75	4.00	3.67	3.67	3.67
Student 2 pre	4.00	4.17	2.80	3.80	2.92	3.50	2.00	2.67	3.50	3.33	4.67	2.33	4.00
Student 2 post	4.50	5.00	4.20	2.40	3.71	4.50	4.00	3.33	3.00	4.67	4.67	3.67	5.00
Student 3 pre	2.50	3.83	4.20	2.60	2.63	1.00	3.33	2.67	3.50	2.67	2.33	3.00	2.33
Student 3 post	3.00	4.50	4.00	2.00	3.29	2.50	4.00	3.67	3.00	3.67	2.33	3.67	3.33
Student 4 pre	2.38	4.83	5.00	3.80	2.44	2.00	2.67	2.33	2.75	2.33	2.00	3.33	2.33
Student 4 post	3.50	4.67	4.40	3.60	2.94	2.00	3.00	3.00	3.75	3.00	3.00	3.33	3.00
Student 5 pre	2.88	3.33	3.80	4.80	2.23	2.00	1.33	3.33	2.22	3.00	3.67	1.33	2.33
Student 5 post	4.38	4.50	4.80	2.40	3.35	3.50	2.33	4.33	3.25	4.00	4.00	3.67	3.33
Student 6 pre	2.88	3.83	2.80	3.80	2.79	4.00	3.33	1.33	2.50	3.00	2.33	4.67	2.33
Student 6 post	3.75	4.17	3.20	3.40	3.42	4.00	3.33	3.33	3.00	4.00	2.67	2.67	3.33

All-time 1, and time 2 measures in the table above are given in accordance with the following sub-scales number-indicators: 1, Goal-setting^*^; 2, Self-efficacy^*^; 3, Time-management^*^; 4, Worry^*^; 5, Psych. skills^*^; 6, Imagery^*^; 7, Arousal-regulation^*^; 8, Concentration^*^; 9, Self-control^*^; 10, Self-observation^*^; 11, Self-evaluation^*^; 12, Perception of prog^*^; and 13, Coping^*^.

### Qualitative findings

The qualitative findings based on individual thematic case analysis were ultimately cross-case analyzed searching for diverging and converging trends in the overall data. Findings revealed both collective trends, as well as individual differences between the participants.

#### Starting point

At the beginning of the program, all participants were assessed filling out questionnaires that were followed up by semi-structured interviewing. Moreover, six key themes were found across the data set. The first theme identified was *general goal setting*: All the participants stated that they set either general or no goals for themselves.

S3: “I sometimes set a general goal for myself that within a month time I need to be able to do this and this…I then just pick up my instrument and see in the moment what to practice, I do not have any other plan…”

S4: “I have never been consistent in goal setting. I have never set concrete goals, but I have tried to be consistent, but without success previously. I struggle to set concrete goals gaining feedback about how I am approaching and developing.”

S2 (string student) and S3 (woodwind student) were organized and structured in their approach to instrumental practice. However, they still expressed a need of becoming more explicit and concrete in their instrumental practice. S1 (jazz student) stated that he was never involved in goal setting whatsoever. S1's practice activity was generally reactive and affective resulting in frustration and procrastination. However, when approaching important concerts, gigs etc., S1 managed to somehow pull himself together.

S1: “Sometimes it might be a vague idea that I want to manage something in the music…I would like to manage this…and that is it…This is usually when I struggle and give up…On other occasions, especially when there is a project that expects me to be prepared and manage so and so…I just do it…”

The second theme found across the data set was *goal insufficiency*: Initial findings revealed that the students did not have any previous experience or knowledge of how to set goals for themselves. This sort of reactive planning seemed to have negative effect on the students' *concentration, satisfaction, self-efficacy*, and *coping* in the face of failure (see **Model 2**). Several of the students reported that they tended to give up when not succeeding immediately.

S4: “When I come to lessons, I often get the impression that I have wasted time and my teacher tells me to do it differently. I feel that my level varies a lot and that I am never able to predict whether my playing will be successful or not.”

The third theme identified across the cases was *physical pain*: All the participants reported that they had experienced (S1, S2, and S3), or currently experienced (S4, S5, and S6) physical pain due to practicing. Consequently, participants had started to manage their sessions in terms of time.

S1: “it has never been a routine for me to chunk up my practice sessions, it is always all in like…Smash…I might be very inspired, or it becomes a habit…no, it becomes a black hole that just takes a lot of energy and motivation. When I finish, I almost do not know what I have actually done, and it seems meaningless. This is what I want to detach from. Previously I used to practice many hours in a row. I stopped when I realized that I needed to eat. This kind of excessive practice made me feel physically unwell.”

S4: “I have unfortunately developed some kind of elbow inflammation. This was due to tense practice, but this has improved…However, 20 minutes might sometimes feel too much. I often tend to practice regardless of pain in my elbow…This also depends on how much I have to practice of course…”

It turned out that this issue was linked to both lack of goal specificity, task relevant focus, and *outcome-orientation*. Several of the participants had an excessive desire to play the music flawlessly straightaway. The discrepancy between the desired level and the actual level of performance caused unnecessary physical and mental tension (see **Model 2**).

The fourth main theme identified was *lack of concentration*: All the participants complained about lack of concentration (e.g., negative thoughts, thought wandering, use of smart phones, tiredness etc.). This turned out to be a general issue. The analysis showed that there was a link between general goals and lack of concentration (see **Model 2**).

S3: “I notice that my head starts to wonder a lot when I practice. It might be what I want to have for dinner, what party to go to etc. It is rather mixed. Some sessions are really consistent and others tends to be worthless…This annoys me a lot…especially that my concentration just wanders away…”

S5: ”The main issue for my low practice efficacy is mind wondering. I tend to think about all the stuff I need to do on that particular day…”

Finally, some of the participants (S1, S4, S5, and S6) expressed that they felt particularly non-volitional (i.e., *volition* was the fifth main theme identified). S1 and S6 expressed that they tended to give up difficult practice tasks due to experiencing failure and lack of volition.

S4: “I am often tempted, having an urge to practice everything in a fast tempo…But sometimes I manage to avoid this by setting the metronome on a slow pace…On the other hand I am often tempted to practice the musical expressions before I actually master the technical foundation of the piece.”

S5: “I have never liked to practice difficult parts. I tend to be impatient, wanting to master difficult stuff immediately, but this is not possible (laughing), thus I feel unsecure about these difficult parts because sometimes it works and others not.”

It turned out that the non-volitional participants were all very eager to achieve a satisfactory outcome as soon as possible. This caused a type of “hastened” practice style based on an overwhelming desire to master the work immediately (see **Model 2**).

In contrast, both S2 and S3 showed inclination toward volitional practice habits.

S2: “Especially if you know the pieces that you are playing very well, I notice that one easily becomes inpatient and one starts to rush the process prematurely. However, all in all, I would say that I am rather conscious about that potential danger and seldom fall into that trap…Sometimes I actually do play pieces prematurely because it amuses me…”

S3: “I really hate to be technically sluggish…Then I prefer to play things way to slow rather than too fast.”

Moreover, the participants turned out to have both shared and individual needs according to the initial assessment. The thematic map (**Model 2**) below illustrates the relationships between the themes identified in regard to the participants' pre-intervention practice routines. The model demonstrates how the themes were intertwined. Forethought processes (i.e., strategic planning, personal beliefs) were linked to the other phases in Zimmerman's cyclical model (i.e., concentration, outcome-orientation, coping, volition) (see Figure 2).

#### Introducing psychological skills

During the first week of implementation, all the participants started to work on hierarchical goal setting. Long-term goals were chunked down to outcome goals, which in turn were chunked down to medium and short-term goals (i.e., weekly and daily goals). Thematic analysis revealed that goal setting was inextricably connected to concentration (see **Model 3**). Participants expressed that goal setting had made things more predictable. Goal setting helped them to become more focused on the task at hand. They discovered the larger “picture” of their instrumental practice. This facilitated a rationale for *why, how* and *what* to practice within each practice session.

S2' diary: “I have now made a strategy with very specific practice today. I have noted various passages that I wish to work on with additional comments on how to pursue. This actually became a tremendously fine day of practice. I managed to be focused for 4 hours and a half …I am very satisfied.”

*S3' diary: “feels like I have a lot to do, and I feel stressed out, no motivation to practice. After having written down and planned everything, I feel motivated again*
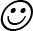
*.”*

S4: “I find setting goals to be very helpful, both in the long run and especially in the short run. Goals positively affect my focus a lot. I notice that my focus decreases a lot when not setting goals. I also manage to keep my focus on one task, instead of being in a chaos of multiple tasks that have to be accomplished.”

*Metacognition* and *self-observation* were themes identified in relation to planning and goal setting (see **Model 3**). The below quotes exemplifies how the students became increasingly metacognitive and self-observant in regard to their instrumental practice.

*S3' diary: “Techniques that really work: When one has too much at the time: Get it written down on paper. Practice repertoire you manage, rather than giving up due to stress. Plan what and how you are going to practice, giving yourself space in order to fulfill it*
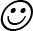
*. Always plan for yourself before you practice. This works much better than trying to plan while actually playing. This enables greater overview of how much time you should spend on each piece.”*

S5' diary: “I am not as concentrated as yesterday. This does not mean that my practice was poor. This is the feeling I have now. I believe that my low concentration is due to old habits of poor self-discipline, and lack of concrete goals. Today I set vague goals. As a result, my practice suffered from too little variation., my brain got fed up. In other words, I need to be better at stopping unwanted thoughts while practicing, and better at practicing in a varied way.”

Some of the participants (two jazz and one string player, S1, S5, and S6) found it arduous to apply the practice journal continuously. However, they found it more motivating and developing to apply the journals, rather than not applying them. E.g., S1 was very persistent writing down goals for himself during the first weeks of the intervention. However, he found it increasingly difficult to keep up with the detailed goal setting. As a result, he preferred to organize his goals mentally. This changed throughout the program as the participant took the initiative to write down his goals once again.

As mentioned, lack of concentration was identified as a main theme among all the participants. In dealing with concentration issues, *centering* was one of the concentration techniques that the participants applied. S3 (woodwind player) did the *centering* every day and expressed that this made him enter the right physical and mental mode when practicing:

S3' diary: “I had a very good practice session! I kind of lost a little concentration in the middle of it. However, then I sat down and decided for myself, “you shall manage this today!). Thus, I did a new centering. This made me perfectly concentrated throughout the whole session.”

As the program progressed, it turned out that three string players (S2, S4, and S5) and one jazz player (S1) were generally preoccupied with not living up to their own and other's expectations (i.e., perfectionistic concerns). This was further unfolded through tense and aroused styles of performing. In dealing with this, the participants spent time on building a non-judgmental sort of focus. This increased the participants' resilience when facing failure. The following sample illustrates how this work was carried out:

Research journal: “*S4 played the beginning of difficult romantic concerto complaining that she always felt stiff and uncertain from the outset. We talked about sensations and how she felt physically while playing. Afterwards I asked her how tense she felt on a scale from 1 to 10, (i.e., 10 being the most aroused state). She felt that she was an 8 when she first played through. We realized that she was very preoccupied with controlling her playing, her sound, technique and perfection. I then asked her if she could try something unusual, abandon all sense of control and accept all mistakes while performing the difficult passage. When she approached the most difficult part of the passage, she made a mistake. The mistake was automatically corrected in a tense manner. She was clearly dissatisfied. As an exercise, we did this once again. The second time I told her to accept all the mistakes and instead pay attention to feeling physically comfortable. This turned out to be difficult, especially when playing in front of the others. However, after a while she mastered this type of non-judgmental focus, thus managing to free herself.”*

This kind of acceptance training became a key-factor influencing all the participants (i.e., especially the four participants who turned out to have perfectionistic concerns). Moreover, the participants actively started to apply new mindsets to their performances (for review see Model 1):

S4' diary: “Audition today, I am really nervous. My goal today was to actually apply the good feeling that I have been working on and to maintain a non-judgmental focus while performing. It really worked!!! That is cool! Afterwards I noticed that there were a few places that were a little out of tune. However, the technique felt fine and I had a comfortable feeling in my body. My technique did not suffer as it usually does while being nervous. In addition, I felt that I managed to maintain a good focus throughout the whole performance. If I could play a few more concerts with this mind-set, I am sure that I will manage concerts much better in the future. To experience this, was much more important than winning or loosing. I see this as a positive part of my development. I practiced a little afterwards, but only one hour, which also made me more focused.”

*S2' diary: “I feel a little nervous about the performance on Thursday. However, when I start thinking rationally, things are just fine*
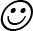
*. However, when the nervous feeling arrives, it kind of tickles…I now accept this feeling and take it for what it is*
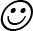
*.”*

S5' diary: “I am very satisfied today as well. I feel that things are fun and not heavy. The reason is because I have finally understood that I should not control my fingers and arms, but let them do the job. My focus is not always top notch. However, the fact that I notice progress makes it much easier to play, which is a good thing.”

**Model 1:**
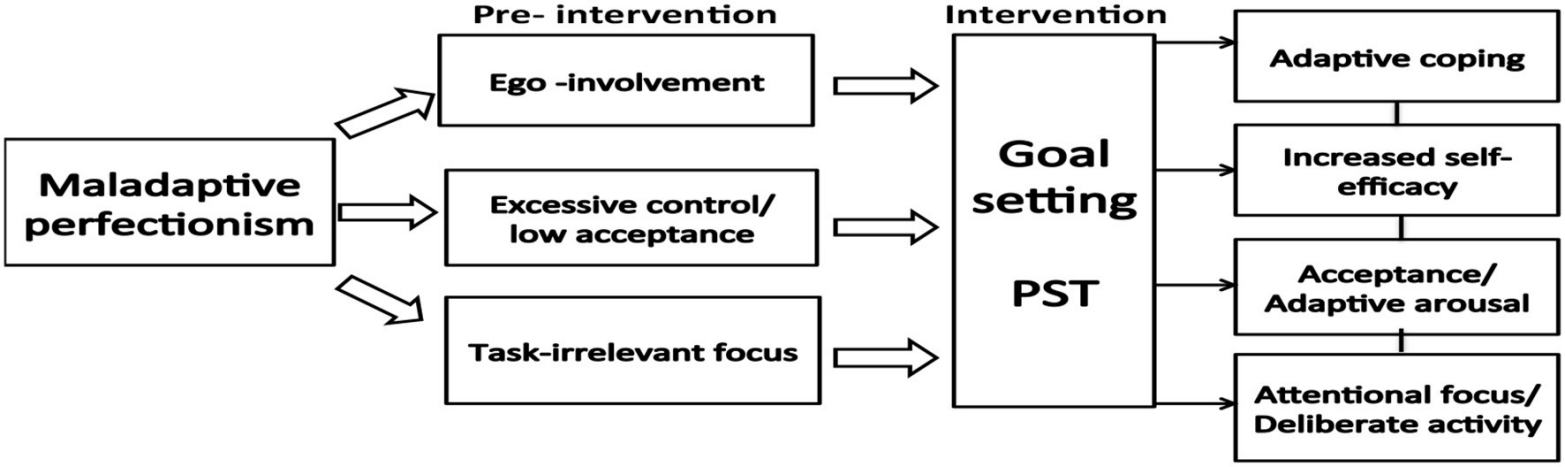
**Overview of themes deriving from the thematic analysis (maladaptive perfectionism)**.

Moreover, *excessive control* was identified as a key issue that debilitated the participants' approach to instrumental practice and performance. Acceptance training (i.e., non-judgmental focus, letting go of control, accepting mistakes without freezing up) facilitated the participants' confidence and understanding of healthy practice. In fact, all six participants reported that they were very satisfied and felt a lot more confident both during dress rehearsal and at the final concert performance. Approaching the dress rehearsal, the participants learnt to change goal focuses from task related practice goals (i.e., focusing on solving concrete practice-related issues) to performance-related goals (i.e., focusing on musical expression, acceptance, letting go in the face of failure etc.). Thus, once automation was attained, the participants learnt to set performance-related goals. In relation to mock performances, the participants integrated pre-performance routines. These psychological preparations turned out to make the participants more resilient, self-efficacious and satisfied with their performance (see Model 1).

S2 diary: “I played through at the concert and I felt that the pianist was still too slow. This made me a little unfocused in the beginning. In any case, things worked much better further out in the piece while playing, I actually felt for the first time ever, that I really managed to solely focus on the music and the expression. I won't even say that I focused on the music. The playing just happened without any thoughts, that was a great feeling.”

S5, who performed a virtuoso piece for violin had increasingly become more confident during the last 4 weeks of the intervention. She finally dared to express the music freely, letting go of unnecessary control. In general, each participant was amazed by the progress of other participants. Having multiple opportunities to apply PST in various settings seemed to have facilitated this progress. Although the participants worked on different issues, they all benefited from discussing and observing how others were working during group sessions.

#### Ups and downs

Throughout the intervention, the participants experienced both ups and downs including optimistic days, as well as days filled with struggle and agony.

S3 diary: “I overestimated my shape. I am not ready for that amount of practice, step by step. Be aware of the fact that you cannot play that much in a row!!”

S5 diary: “Today was a heavy day of practice. I focused too much on technique, did it wrongly and felt that I did not manage to accomplish my task. This resulted in my shoulder aching. I believe that most of the things that did not work out could have been avoided by having another focus/attitude. Some days are just like this. I have played a lot lately, chamber music, lesson and practice. I should probably have taken a break today. I will use this drawback as an opportunity to work better tomorrow.”

In general, writing down experiences (i.e., positive and negative ones) tended to foster self-awareness in the participants. This enabled them to self-regulate their subsequent actions. In the face of failure, the participants seemed to attribute their struggles to inappropriate use strategies. Moreover, adaptive coping was identified as a key-theme based on proactivity and metacognition (see **Model 3**).

#### Experiencing the program

Interviews regarding participants‘ experience of the PST revealed that all participants gained more knowledge about themselves as music performers and practitioners.

S1: “Generally, I am now focused toward fundamental basics in my playing and how to develop further. Instead of wasting time on thoughtless practice, I have become better at identifying exactly what is needed to accomplish specific tasks in each practice sessions…It's like killing darlings.”

S2: “Now I recognize what I really need in order to accomplish what I am aiming at. In essence, to realize when I function the best, and when I have time and energy. It also entails preparing my head in order to accomplish what I am searching for. I now find instrumental practice attractive and energizing, rather than a nightmare…(laughing.”

S4: “The PST has been more rewarding than I could initially imagine. I have become much better at both practicing and performing. Now I have more confidence in myself and in what I am doing compared to before…In addition, my process of practice has changed a lot, even though I really did not believe it would at the beginning of the programme…I really did not know that there was an alternative. One really does not speak about these issues at the academy.”

Questions assessing participants' perceptions of electronic practice journals revealed that some of the participants liked them more than others. However, all participants expressed that the journals had made them more aware and reflective in regard to instrumental practice. S6 stated that she was not interested in using gadgets. She preferred writing comments on paper, which she did accordingly. S3 had similar experiences using the practice journal. However, he had recognized the usefulness of continuing this practice.

S3: “One thing that has been very helpful is to write diary about my practice making some notes from day to day, what I shall do the day after. In the beginning, I wrote down more details than I do now because it has become automatized. However, now I would like to restart to be more detailed once again, because I notice that the exercises tend to become thoughtless without writing down goals.”

Although it only took an extra 10 min a day, the actual deed of getting oneself to write in the diary seemed to require extra effort and volition.

S4: “…it is not exhausting while one is actually doing it. It is to motivate oneself to actually write down goals and reflections. All in all, it was worth the extra effort, because this was the only way that I could actually understand the depth of my practice!!”

The four participants involved in perfectionistic concerns (S1, S2, S4, S5), turned out to have become more task-oriented, resilient, adaptively coping, and self-efficacious in their practice and performance (see Model 1).

S2: “Well, I would say that the main difference consists of not being too egocentric before concerts. This is not about me, and how great I want others to believe that I am to play the instrument. It is all about being satisfied and accepting that one is in the middle of a process that takes time, and that every outcome is just fine. It's manual work, it's all about making things work…I feel that I have become much more harmonic about both my practice and performance. It is actually not that deadly serious anymore, it is not Armageddon or earthquake when one performs…On the other hand, I emphasize the fact that I am grateful to be able to express something musically that others might appreciate, I have become more aware of this fact.”

Furthermore, post-interviews demonstrated that goal setting enhanced the participants' motivation, concentration and self-efficacy (see Model 2).

S6: “I notice that I become motivated by perceiving that I am more structured and disciplined…The more detailed my practice goals, the better practice and concentration.”

S4: “The main difference is that I believe in what I am doing to a greater extent. I feel that I make better progress, which also makes me more happy in general.”

**Model 2:**
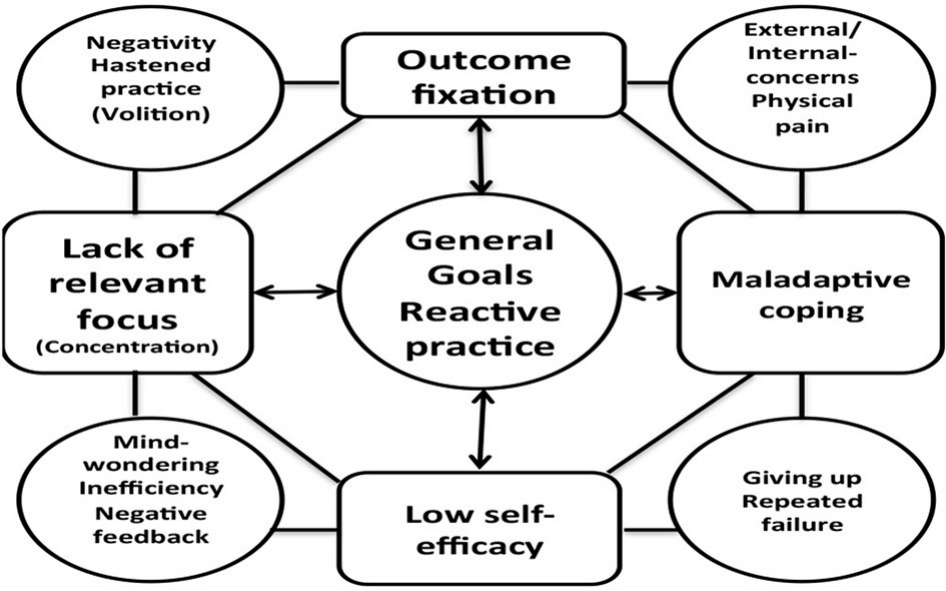
**Themes associated with practice habits prior to intervention**.

In general, all participants perceived the program as personally developing. Thus, the PST facilitated their motivation and joy in making music. The results from the thematic analysis were mapped down in thematic charts:

Model 1 illustrates themes based on work with perfectionistic concerning participants (S1, S2, S4, and S5). Model 2 illustrates interrelations among themes generated from the data regarding pre-interventional practice habits. Model 3 illustrates interrelations among themes based on program efficacy.

**Model 3:**
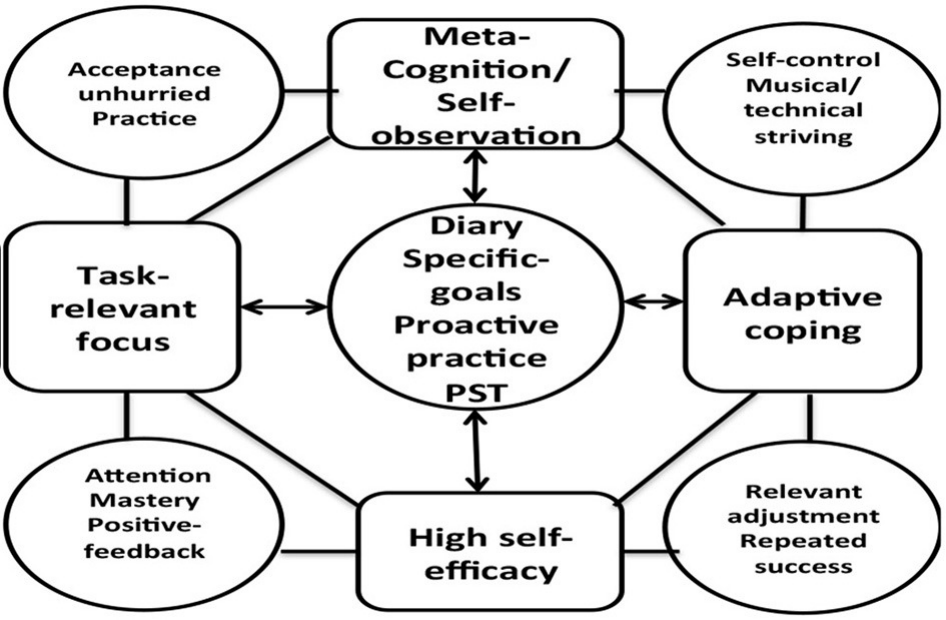
**Themes associated with practice habits during and after intervention **.

#### Follow-up interviews

Follow-up interviews were conducted 8 months after finishing the intervention. Generally, the interviews revealed that the participants were still actively applying psychological skills acquired during the program. Firstly, all the participants expressed they had become generally more aware and self-reflected in their daily practice.

S1: “My practice is better than it has been in many years…I now chunk up my practice. My practice is to a much greater extent related to concrete, practical goals and sub-goals that I grasp easily. This makes my mind more balanced during practice. In other words, I do not come out from a black hole every time I put down the instrument. My practice is less victim of trying to make mind-blowing music every time I approach the instrument.”

S2 (string player) stated that she still struggling with some self-criticism prior to concerts. However, being aware of how to deal with it helped significantly. S2 had continued to use imagery and acceptance training in her pre-performance routines. However, she expressed a need of becoming even better at applying PST more frequently. Finally, she could not imagine herself practicing without the knowledge and use of the techniques.

S3 (woodwind player) continued to be specific in his planning and expressed that he managed to maintain his concentration applying centering. He had become much more resistant due to being patient and volitional. Additionally, S3 had become more involved in self-evaluation and self-observation through recording himself during practice sessions. Finally, S3 uttered a continued need for individual PST sessions.

S3: “I have to say that I really miss having a coach with whom to discuss problems and challenges, a coach who is not my instrument teacher. I would have loved to continue this work throughout the rest of my time here at the Academy.”

S4 (string player) was still applying psychological skills. S4 expressed a need of continuity.

S4: “I am not applying the practice journal as frequently as before. I notice that this affects my focus negatively. My concentration was better during the PST-intervention.”

However, S4 also stated that she had become more volitional and deliberate as a result of the PST intervention. Moreover, she had become more self-confident and resilient in the face of failure.

S4“I have progressed a lot lately. I feel more secure and I play more in tune with a bigger, more sonorous relaxed sound. I am much more successful playing more difficult pieces. I have started to find a serenity and a proudness in what I am doing.”

S6 (Jazz player) was actively applying a practice journal writing down specific goals.

S6: “to work in a structured way makes me motivated. In the past, I believed that structured practice was monotonous and dull. However, I now experience that my practice is much more releasing! I have a clear perspective of what I want to practice every day. This has made me less preoccupied with what sort of musician I am becoming. I now know this will suddenly appear as a result of what I love doing at the moment.”

Compared to before, S6 recognized that she now noticed daily progress. In general, all the participants completing the follow up interview were eager to continue applying psychological skills in their practice and performances^6^.

## Discussion

The aim of the present article was to investigate the impact of a PST-intervention for musicians from a self-regulated learning theory perspective. A mixed method approach including qualitative measures corroborated by quantitative measures operationalized the study. The present study found that the participants were reactive practitioners to various degrees. Themes identified included general goal setting, concentration, volition, physical pain, and perfectionism (see Models 1, 2). Furthermore, the study found that PST (with special emphasis on planning and goal setting) facilitated cyclical self-regulated learning patterns (For review of SRL see Figure 2). In essence, the PST was found to be associated with increased concentration, self-observation, self-efficacy, and adaptive coping in the face of failure (see Model 3). Finally, the PST-intervention reduced the participants' level of worry and anxiety in performance situations (see Model 1). An 8-months follow up interview revealed that the participants were still actively using psychological skills. The qualitative results were generally supported by the quantitative results (see Tables 1–3, and Figure 3).

### Planning and goal setting

In accordance with previous studies, the present study found that the participants were uninvolved with deliberate planning of instrumental practice (Jørgensen, 1996; Hatfield et al., 2016). The participants had little experience setting specific goals for their instrumental practice. Thus, they applied either general goals, or no goals at all in relation to instrumental practice. This seemed to make the participants self-inefficacious and worried about performing in front of others. Zimmerman (2008) addresses how lack of specific planning and goal setting negatively affects self-efficacy, motivation, and ability to cope due to task-irrelevant focus. This seemed true for the present study. All participants complained about difficulties in maintaining attentional focus. Quantitative findings indicated a general increase in goal setting from time 1 to time 2. Accordingly, qualitative findings revealed that specific and hierarchical goals enhanced the participants' concentration, self-observation, and self-efficacy for instrumental practice and performance. Research investigating the role of specificity and hierarchical goal setting has found that people become more self-observant, self-efficacious, volitional, and concentrated during learning when applying such goals (Schunk and Rice, 1989; Schunk, 1990; Locke and Latham, 2002). As the participants approached the final concert, pre-performance routines (i.e., targeting the outlook of the performances) were applied. During this stage, the participants worked on personal performance cues that emphasized the musical messages they wanted to convey to the audience. Locke and Latham (2002) distinguish between learning tasks and performance tasks in skill acquisition (i.e., the former is related to process goals, and the latter to performance goals). Zimmerman and Risemberg (1997) and Zimmerman and Kitsantas (1999) found that shifting from process goals to performance goals enhanced performance. In essence, as deliberate task oriented practice is carried out, layers of correct motor-control are accumulated. The result is automatized skills (Zimmerman and Kitsantas, 1999). Moreover, becoming conscious of this process seemed to facilitate effortless performance and joy in the participants. Opposite patterns were found in relation to pre-interventional practice habits. Motivated participants lacking proper involvement in forethought phase activity (see Figure 2) tended to seek immediate mastery. This outcome-oriented practice had a negative impact on the participants' self-efficacy, worry, and coping. In essence, some students responded to mistakes by altering their effort. This sort of maladaptive reaction seems to be particularly unfortunate in regard to over practice and injuries (Lemyre, 2005).

### The role of perfectionism

The quantitative findings revealed that all participants decreased in worry after implementation of PST. Additionally, quantitative findings revealed that the participants' ability of regulating their arousal increased from pre- to post-test. These measures fit the participants' perception of becoming increasingly more resilient, self-efficacious and confident in performance situations. Having multiple opportunities to apply psychological skills in front of others generated a learning environment that enabled *vicarious learning, social persuasion, mastery-experiences*, and *physical/psychological certainty* (i.e., the four key sources of self-efficacy) (Bandura, 1977). This instigated self-efficacy for performance and practice in the participants. The effect size measures indicated that self-efficacy was non-significant. However, further investigation demonstrated that this was due to insufficient statistical power. Two of the participants turned out to have high and almost similar scores on both time points. This biased the effect sizes due to a small sample size. The remaining four participants had a significant quantitative increase in self-efficacy between the two time points.

Perfectionistic concerning participants became perfectionistic strivers as they progressed throughout the program. In line with Stoeber (2012), they learnt to tolerate imperfection and mistakes, thus worrying less about social approval. Instead of avoiding negative thoughts, the participants recognized and accepted them. The participants started to view performance situations as unique possibilities to try out the psychological skills they were working on. This made the participants increasingly more resilient (Hayes and Strosahl, 2004; Stoeber and Eismann, 2007). Within Acceptance and Commitment Therapy (ACT), this approach of acceptance and non-judgmental focus has revealed to be highly effective (Hayes and Strosahl, 2004; Russel, 2006). Correspondingly, *The Inner Game of Tennis* advocates that a non-judgmental focus is needed in deliberate practice and performance, accepting whatever outcome (Gallwey, 1997).

### Sources of volition, awareness, and self-reflection

The use of practice journals greatly facilitated the participants' self-awareness, metacognition, and self-observation. Furthermore, the students suddenly had the means to plan and reflect on their practice without being “tempted” and affected by their instrument and spontaneous desires. In fact, the temptation to start out practicing in a random intuitive way turned out to be a source of distraction in several of the participants prior to intervention. In essence, the participants turned out to be more volitional and satisfied with their practice due to ending and starting every practice session writing down reflections and goals. Paradoxically, a striving toward peak performance seems to make music students particularly prone to reactive instrumental practice. Instead of deliberately analyzing what is needed on the path to perfection, reactive learners attempt the final outcome in an overenthusiastic way (Weinberg and Gould, 2011). In line with previous research, the present study revealed that the participants' coped adaptively when facing failure due to having become more aware and metacognitive (Zimmerman, 2008). In essence, when the participants met obstacles, they reflected on how to solve them in the future. Consequently, when they identified precisely *what, how*, and *why* they succeeded or failed, they became more certain about how to continue the activity. Attributions are more likely to emphasize use of strategies, rather than lack of personal ability when recognizing the real cause of success and failure (Weiner, 1985). Accordingly, recent research, has found that self-regulated music students were more likely to be self-efficacious and proactive in their planning of instrumental practice. In essence, the study confirmed cyclical self-regulated learning in self-regulated music students (Hatfield et al., 2016).

Drawing on a previous pilot-intervention study (Hatfield and Lemyre, 2016), the present intervention study constituted an individual focus. Thus, each participant's personal needs were closely followed up. In contrast to previous psychological skills interventions for musicians (e.g., Hoffman and Hanrahan, 2012; Osborne et al., 2014), the present study's long-term individual focus is believed to have accelerated personal growth and progress throughout the program. It is limited to which extent one is able to learn, apply and implement psychological skills when depending on a few weeks of training (Andersen, 2000; Hays, 2009). The length of the present PST program enabled internalization and active appliance of the skills. Accordingly, follow up interviews demonstrated that the present study enabled long-term self-regulatory development. Clark and Williamon (2011) found that participants requested more precise application of the mental skills that were applied in their study. In addition, the participants requested both better integration of the mental skills in various performance situations, and greater use of group discussions (Clark and Williamon, 2011). These findings became valuable for the present study, and were, accordingly, considered during the making of the present PST-intervention.

The study was subject to some limitation. Firstly, the number of participants was very small.

This had implications for generalization of the results and the quantitative methods applied. A greater amount of cases (>30) would have enabled external validity. Second, the present study did not include a control group. Having a control group might have strengthened the overall understanding of the effects comparing results of the experimental group and the control group. Accordingly, this would be recommended for future interventions.

## Conclusion

The present study presented a vast variety of empirical data targeting benefits, challenges, and implications of a PST-intervention for musicians. The main body of findings derived from qualitative sources including participants' practice journals, semi-structured interviews, and research logs. Qualitative findings were supplied by quantitative measures indicating the effects of the intervention. Moreover, the overall design attempted to cover both the breadth and depth of the intervention.

In line with self-regulated learning theory, the present study found that forethought phase activity (i.e., strategic planning, self-assessment and goal setting) facilitated performance phase and self-reflection phase activity (Zimmerman, 1989, 2002; Hatfield et al., 2016). Initial self-assessment (i.e., identifying personal strengths and limitations) enabled the participants to set goals that corresponded to personal needs. The application of practice journals enabled self-regulated learning and the organization of psychological skills. Although some of the participants found consistent use of practice journals challenging, extra effort invested clearly paid off. Implicationally, the appliance of PST is highly recommended as it enables resilience, confidence and self-regulated learning in musicians. Accordingly, providing music students with the opportunity to discuss personal practice-related issues and frequently perform in front of other students is believed to enrich the curricula in higher music education. Moreover, it is beyond doubt that PST has the potential of facilitating music students' personal development, satisfaction and motivation.

## Author contributions

The author confirms being the sole contributor of this work and approved it for publication.

### Conflict of interest statement

The author declares that the research was conducted in the absence of any commercial or financial relationships that could be construed as a potential conflict of interest.

## Footnotes

^1^*Perfectionistic striving* is characterized by a focus toward high standards, tolerance for imperfection and mistakes. *Perfectionistic concerners*, concern about mistakes, negative feedback, and approval/non-approval from others. Perfectionistic concerners are worried about potential discrepancy between personal expectations and performance (Stoeber, 2012).

^2^*Attentional focus (AT)* contains the following: (a) Selective attention (i.e., identifying the relevant cues for each task) (b) maintenance of AT over time, (c) being aware of the situation and performance errors, and (d) Shifting focus when necessary. *Arousal-regulation* refers to the capacity of regulating physical and mental arousal in accordance with situational demands. Applying *imagery*, one can recreate and memorize previous positive experiences, and/or one can picture and imagine new situations to mentally prepare for performances. *Acceptance training/self-talk* aims to building self-confidence. Athletes who are insecure tend to avoid doing mistakes, while self-confident athletes are willing to take chances during competition (for further review see Weinberg and Gould, 2011).

^3^Building on the pilot study (Hatfield and Lemyre, 2016), an emphasis toward satisfying participants basic psychological needs (see Deci and Ryan, 2000) was prioritized throughout the present study. However, this was not reported explicitly as this would have been beyond the scope of the present study.

^4^The recruitment questionnaire applied “time for individual practice,” “availability and interest in PST” as inclusion criteria. “Work besides studies” (unavailability) “previous experience with PST” were the exclusion criteria.

^5^Hedges' G test of standardized effect size is especially recommended for small samples as it adds a correction factor for small samples.

^6^S5 had quit music studies. Consequently, the follow up-interview was not completed.
